# Characterization of adherent primary cell lines from fresh human glioblastoma tissue, defining glial fibrillary acidic protein as a reliable marker in establishment of glioblastoma cell culture

**DOI:** 10.1002/cnr2.1324

**Published:** 2020-11-30

**Authors:** Susanne Grube, Diana Freitag, Rolf Kalff, Christian Ewald, Jan Walter

**Affiliations:** ^1^ Department of Neurosurgery, Section of Experimental Neurooncology Jena University Hospital – Friedrich Schiller University Jena Jena Germany; ^2^ Department of Neurosurgery Brandenburg Medical School Brandenburg Germany; ^3^ Department of Neurosurgery Clinical Center Saarbruecken GmbH Saarbrücken Germany

**Keywords:** GFAP, glioblastoma, in vitro, primary cell line

## Abstract

**Background:**

Primary adherent glioblastoma cell lines are an important tool in investigating cellular and molecular tumor biology, as well as treatment options for patients.

**Aim:**

The phenotypical and immunocytochemical characterization of primary cell lines from glioblastoma specimens during establishment is of great importance, in order to reliably identify these cell lines as primary glioblastoma cell lines.

**Methods and Results:**

Sixteen primary adherent cell lines out of 34 glioblastoma samples (47%) were established and further characterized. For phenotypical characterization, morphology and growth characteristics of the cells were classified. The cell lines had a high growth rate with a doubling time of 2 to 14 days. Morphologically, the cells displayed spindle‐form or polygonal to amorphous shapes and grow as monolayer or in foci without evidence of contact inhibition. The cells were able to migrate and to form colonies. For further characterization, the protein expression of the astrocyte‐specific protein glial fibrillary acidic protein (GFAP), the glial marker S100B, the neuronal marker TUBB3, and malignancy marker VIM, as well as the progenitor markers NES and SOX2, the proliferation marker MKI67, and the fibroblast marker TE7 were determined. Based on the immunocytochemical validation criterion of a coexpression of GFAP and S100B, 15 out of these 16 cell lines (94%) were defined as primary glioblastoma cell lines (pGCL). All 15 pGCL expressed TUBB3 and VIM. NES and SOX2 were stained positively in 13/15 and 6/15 pGCL. MKI67 was expressed in 11/15 and TE7 in 2/15 pGCL.

**Conclusion:**

These results point out that in self‐established primary adherent glioblastoma cell lines, the expression of the specific astrocytic and glial markers GFAP and S100B and of the malignancy and progenitor markers VIM, NES, and SOX2 has to be validated. These data show that primary cell lines of glioblastoma origin with high malignant potential are reliably to establish using standardized validation criteria.

## 1 INTRODUCTION

Malignant gliomas are the most common and most aggressive brain tumors in adults with currently no cure,^1^ since they are characterized by pronounced invasiveness, as well as extensive intra‐ and intertumor heterogeneity^2^, ^3^, ^4^, ^5^ and a distinctive chemo‐ and radio‐resistance. The standard treatment regime covers surgery to remove as much of the tumor as possible, followed by combined radio‐chemotherapy.^6^ New data show an additional benefit of an additive effect of alternating electric fields.^7^ Given the overall poor prognosis, there is still a need to develop improved therapeutic options.

Reliably identified and characterized glioblastoma cell lines as a sustainable source of vital and proliferating malignant cells are invaluable for investigating tumor biology or for functional analyses such as response prediction.^8^, ^9^ Cancer cell lines, as the primary in vitro model system, are the standard both for exploring the basic cellular and molecular tumor biology and as preclinical models for testing new treatment modalities. For this reason, there is still a high demand for rapidly available cell models of primary brain tumors that are very close to the original tumor. Cell lines, which have been used widely in neuro‐oncological research, are adherent glioblastoma cell lines established around 40 years ago.^10^, ^11^, ^12^, ^13^, ^14^ It has become increasingly clear, however, that phenotypic characteristics and the multitude of genetic aberrations found within repeatedly in vitro passaged cancer cell lines often bear little resemblance to those found within the corresponding primary human tumor.^15^ Primary cell lines directly derived from operations and then cultivated over as few passages as possible, more closely mirror the primary tumor, than commercially available cell lines do. Adherent primary glioblastoma cell lines provide a versatile and renewable resource to analyze the biology of tumor cells. Tumor cell proliferation, cell death and migration represent potential therapeutic opportunities that are accessible in adherent primary glioblastoma cell lines, and one can screen for agents that selectively and directly target them. This led us to cultivate biologically relevant adherent primary glioblastoma cell lines to define standardized characterization criteria for the validation of these cell lines. We are routinely able to generate characterized adherent primary glioblastoma cell lines. Continuous adaptation of our current technique has improved the processes for cultivation of cell lines directly from fresh glioblastoma tumor samples.

There is some literature available on the establishment of primary glioblastoma cell lines. Unfortunately, phenotypic as well as immunocytochemical characterization using a panel of marker proteins to confirm the identity of established tumor cells is often missing. Often only single primary glioblastoma cell lines are generated for specific analyses but not described in detail. Four work groups^14^, ^16^, ^17^, ^18^ described the establishment and analysis of a number of primary glioblastoma cell lines. However, they did not prove the expression of specific glial markers for these cell lines. Only Mullins et al,^18^ who established 17 primary glioblastoma cell lines, analyzed the marker expression of glial fibrillary acidic protein (GFAP), NES, and VIM in five cell lines using flow cytometry as an example. Among these cell lines, 75% to 95% GFAP‐expressing cells could be detected.

This lack of reliable characterization of self‐established primary glioblastoma cell lines demonstrates the urgent need for an accurate immunocytochemical analysis during cell line establishment. There are several well‐established markers used for diagnosis of glioblastoma, but none of them is solely tumor specific. This limiting factor in the investigation of glioblastoma cells in vitro led us to investigate a panel of different markers for immunocytochemical analysis. This marker panel includes the astrocytic marker GFAP, the glial marker S100B (S100 calcium binding protein B), the neuronal marker TUBB3 (tubulin beta 3 class III), the tumor marker VIM (vimentin) and the progenitor markers NES (nestin) and SOX2 (SRY‐Box 2), as well as the proliferation marker MKI67 (Ki‐67) and the fibroblast marker TE7. The immunoprofile of the glial marker GFAP in glioblastomas is similar to astrocytomas.^19^, ^20^ Other, but less specific astrocytic, markers are S100B and MAP2a (microtubule‐associated protein 2a).^21^ Neuronal markers as beta III tubulin (TUBB3, Tuj‐1), neurofilament protein (NFP), and neuron‐specific enolase (NSE) are aberrantly expressed both in glioblastoma cell cultures and in patient biopsies.^22^ The progenitor markers NES, a cytoskeletal protein expressed during the development of the central nervous system, and SOX2, a transcription factor responsible for maintaining stem cell features of embryonic stem cells and pluripotent stem cells,^16^, ^23^ are expressed under serum‐free culture conditions but also in the first passages of primary cell lines.^20^ Both have been found to be up‐regulated in cancer, including high‐grade gliomas.^23^, ^24^ They promote stem cell features, tumor cell proliferation, migration, and invasion.^20^ As validation criterion, we have defined that all cell lines with an immunopositivity for both markers GFAP and S100B are primary glioblastoma cell lines (pGCL). The other markers selected were used to characterize the malignancy of the established pGCL and to exclude the cultivation of undesired fibroblasts.

Applying the defined validation criteria, we established 15 adherent primary glioblastoma cell lines (pGCL) under serum conditions. Precisely characterized primary glioblastoma cell lines are an important tool in investigating cellular and molecular biology as well as evaluating treatment options for patients with malignant gliomas. The present technical study aims at simplifying the establishment of individual primary glioblastoma cell lines and defines reliable validation and characterization criteria for the establishment of primary glioblastoma cell lines with regard to expression of marker proteins, morphological characteristics, and growth kinetics.

## 2 MATERIAL AND METHODS

### 2.1 Primary cell line establishment

Tumor tissue of 34 glioblastoma patients (22 male, 12 female; mean age: 67 years, range 43‐83 years) was collected during resection and transferred cooled to the laboratory. For cell line establishment, tissue from the border of the tumor was used. Under sterile conditions, it was rinsed in 1x PBS. Obvious vessels, clotted blood, and charred tissue were removed. The explant was grossly dissociated using scalpels and further enzymatically dissociated by incubation with Collagenase Type IVa (250 U/mL, Sigma‐Aldrich, St. Louis, Missouri) and Pronase E (2.5 U/mL, Sigma‐Aldrich, St. Louis, Missouri) in 1xPBS (Thermo Fisher Scientific, Waltham, Massachusetts) at 37°C for up to 1 hour. The cells were centrifuged at 300*g* for 5 minutes at 4°C, resuspended in 1x DMEM supplemented with 10% FBS (Thermo Fisher Scientific, Waltham, Massachusetts) and seeded in a cell culture flask, as described previously.^16^, ^25^ After a 2‐day resting phase, the cell culture supernatant was removed, the cells were washed with 1x PBS (if there was cellular debris in the culture), and a new cell culture medium was added. Change of cell culture medium and microscopic monitoring (phase contrast) was performed routinely every 2 days. This study was approved by the local human research ethics committee of the Friedrich Schiller University Jena (AZ: 3253‐10/11) and was performed in accordance with current legislation and the ethical standards laid down in the 1964 Declaration of Helsinki and its later amendments.^26^ All patients gave their written informed consent to study participation. The samples were collected between 2011 and 2013, hence tumor diagnosis was performed according to the fourth edition of the World Health Organization (WHO) classification of tumors of the central nervous system.^27^

### 2.2 Cell lines

The cell lines A‐172^28^ and LN‐229^29^ were purchased from American Type Culture Collection (ATCC, Manassas, Virginia). The cell line U‐251 MG^30^ was purchased from Cell Lines Service (CLS, Eppelheim, Germany). The cell lines were cultivated in 1x DMEM supplemented with 10% FBS.

### 2.3 Cell line doubling times

Five thousand cells per well were plated in 24‐well cell culture plates and allowed to attach for 24 hours. The total viable cell number was counted in triplicate every 24 hours for up to 14 consecutive days by Trypan Blue exclusion (Sigma‐Aldrich, St. Louis, Missouri) and a hemocytometer. Doubling times were then calculated using an online calculator.^31^

### 2.4 Morphology assessment

Five thousand cells per well were plated in triplicate in 24‐well cell culture plates and allowed to attach and proliferate for 48 hours. After fixation (ice‐cold 70% ethanol for 15 minutes, Carl Roth, Karlsruhe, Germany) and staining with methylene blue solution (Carl Roth, Karlsruhe, Germany), growth type of the cell culture (monolayer, focal growth) and the cellular phenotype (dendritic‐like, spindle‐form, polygonal, amorphous, with or without cellular protrusions) was assessed to divide the cell cultures in subgroups according to their morphology.

### 2.5 Senescence assay

Five thousand cells per well were plated in triplicate in 24‐well cell culture plates and allowed to attach for 24 hours. After fixation, cells were stained overnight for senescence‐associated ß‐galactosidase (SA‐ß‐Gal) as described by Dimri et al.^32^ Senescent cells were quantified by light microscopy. Only strong SA‐ß‐Gal positive cells were counted; it reflects the replicative age of cultivated cells. A proportion of up to 5% is usual for a proliferating cell culture.

### 2.6 Migration analysis

The cells were plated in 24‐well cell culture plates in triplicate and cultivated for 36 hours to achieve confluence. The cells were then synchronized for 12 hours in serum‐free 1x DMEM. In the confluent cell layer, a straight line was scratched using a p10 pipet tip. The cells were incubated for further 24 hours, and images were acquired after 0, 6, 12, and 24 hours from the same field of view. The scratch distance was calculated using ImageJ 1.47v (NIH, Bethesda, Maryland^33^) as mean of 10 horizontal lines drawn over the scratch. Furthermore, the cell cultures were divided into subgroups according to their migratory behavior: no migration, single cell migration, and collective migration.

### 2.7 Colony formation assay

Five hundred cells per well were plated in triplicate in six‐well cell culture plates and incubated over 21 days with regular change of medium. After fixation (ice‐cold 70% ethanol for 15 minutes) and staining with methylene blue solution, formation of colonies was assessed (single cells without colonies—no colony formers, single cells with colonies—weak colony formers, only colonies—strong colony formers).

### 2.8 Immunocytochemical staining

For analysis of protein expression, cells were grown on Poly‐d‐lysine (PDL, Sigma‐Aldrich, St. Louis, Missouri) coated cover slips and stained immunocytochemically. To visualize expression and cellular distribution of different marker proteins, the following primary and secondary antibodies were used: mouse monoclonal anti‐GFAP (Merck Millipore, Burlington, Massachusetts; 1:250), mouse monoclonal anti‐S100 (ß‐subunit) (Sigma‐Aldrich, St. Louis, Missouri; 1:200), rabbit polyclonal anti‐beta III Tubulin (TUBB3, abcam, Cambridge, UK; 1:1000), mouse monoclonal anti‐Vimentin (clone Vim 3B4, DakoCytomation, Glostrup, Denmark; 1:100), rabbit polyclonal anti‐Ki‐67 (H‐300) (MKI67, Santa Cruz Biotechnology, Dallas, Texas; 1:250), mouse monoclonal anti‐Nestin (10C2) (NES, abcam, Cambridge, UK; 1:100), mouse monoclonal anti‐Sox2 (L1D6A2) (Cell Signaling Technology, Danvers, Massachusetts; 1:400), mouse monoclonal anti‐Human Thymic Fibroblasts (TE7) (Santa Cruz Biotechnology, Dallas, Texas; 1:500), goat anti‐Mouse IgG‐FITC, and goat anti‐Rabbit IgG‐FITC (both Sigma‐Aldrich, St. Louis, Missouri; 1:200). Nuclei were counterstained with bisBenzimide H 33258 (Hoechst33258, Sigma‐Aldrich, St. Louis, Missouri; 1 μg/mL). The number of stained cells was examined by fluorescence microscopy and scored as follows: 0, no expression; 1, ≤5% of cells; 2, ≥5% of cells; 3, ≥25% of cells; 4, ≥50% of cells, and 5, ≥75% of cells. GFAP expression was further grouped: 0, no cells; low, ≤5% of cells; medium, >5% to ≤50% of cells; high, >50% of cells. For each cell line, one cover slip per antibody was stained, completely examined and subjectively evaluated. Examples were photographed using an AxioPlan microscope system (Zeiss, Oberkochen, Germany). All cell lines with cells immunopositive for both GFAP and S100B were defined as “primary glioblastoma cell lines (pGCL).”

### 2.9 Statistical analysis

All statistical analyses were carried out with the IBM SSPS Statistics v23 software (IBM, Armonk, New York). Statistical significance was defined as *P* ≤ .05. For the analysis of protein expression levels (immunocytochemistry), Spearman's rank correlation was used. Scratch assay results were statistically evaluated with variance analysis (ANOVA). To further test intergroup comparisons for statistical relevance, the Mann–Whitney *U* test was used.

## 3 RESULTS

### 3.1 Cultivation efficiency and doubling time

**FIGURE 1:**
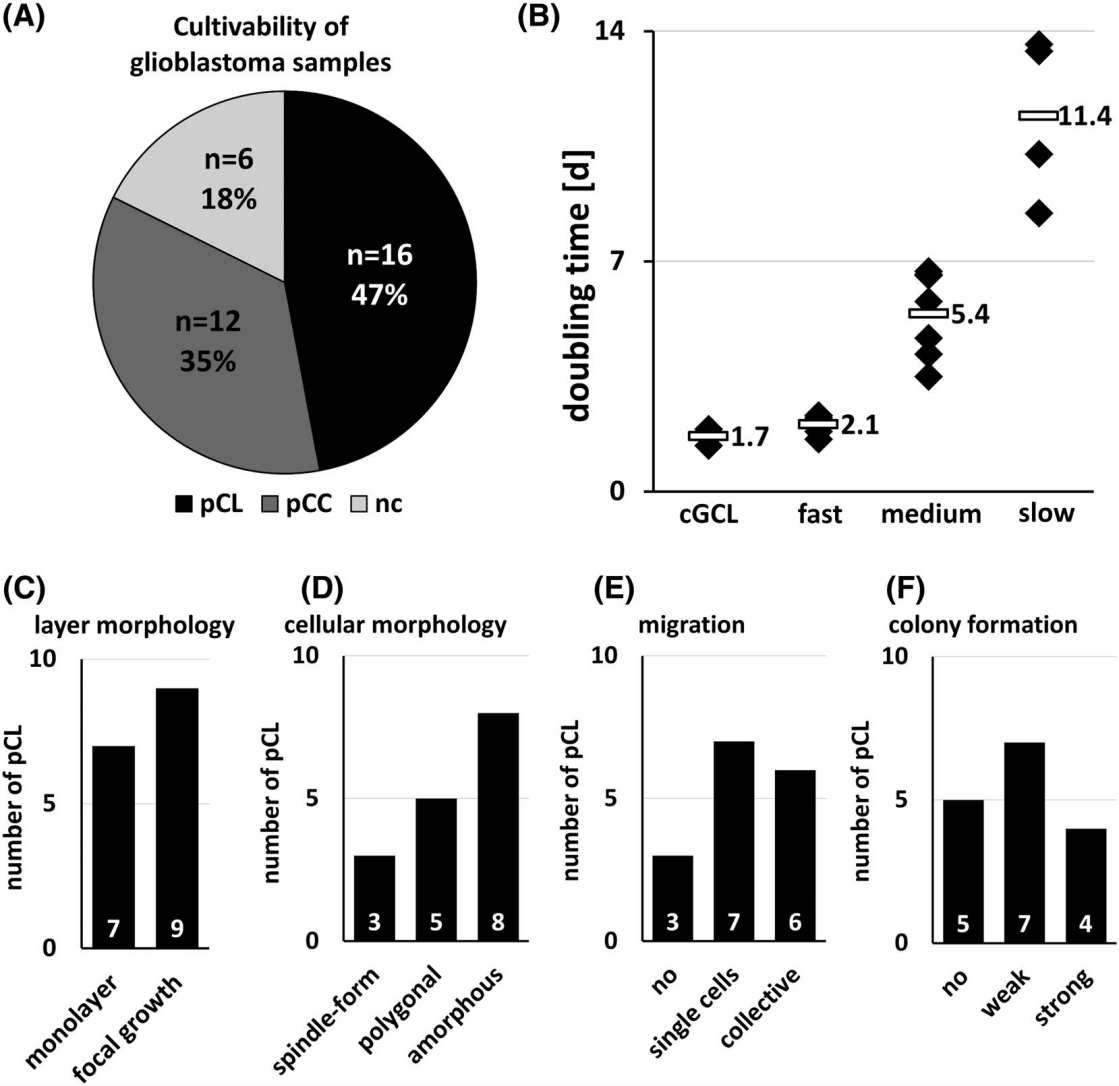
Cultivability of the 34 processed glioblastoma samples. A, Sixteen glioblastoma‐derived primary cell lines (pCL, black) and 12 short‐term primary cell cultures (pCC, gray) were established. Six tumor samples were noncultivable (nc, light gray). B, Doubling time of the primary glioblastoma‐derived cell cultures. Five pCC were fast growing (<3 days), seven medium (3‐7 days), and four pCC slow growing (>7 days), compared to the three cell lines (cGCL, mean 1.7 days). C‐F, Cytomorphological characterization of the 16 pCC. C, Layer morphology (FG, focal growth; ML, monolayer). D, Cellular shape. E, Migratory behavior. F, Colony formation

Out of 34 glioblastoma specimens, we successfully established 16 glioblastoma‐derived primary cell lines (pCL, 47%, Figure 1A and Table 1) with continuous proliferation that could be passaged regularly. Twelve of 34 glioblastoma tissue samples (35%) resulted in short‐term primary cell cultures (pCC), which did not grow further after the first or second passaging and therefore could not be analyzed or stored. The remaining 6/34 tumor samples (18%) did not attach and proliferate after disaggregation (noncultivable, nc). It is assumed that the area of tumor from which the sample originates can cause the differences in cultivability. If the sample was taken in a necrotic area, it is not possible to cultivate cells. If the tumor samples originate from the transitional area between necrosis and proliferating tumor, cultivation is possible, but often such large amounts of cellular debris are found in the culture that the cells die. The presence of erythrocytes during the cultivation of samples with a high blood supply also leads to cell death. Methods for lysis of erythrocytes were not tested in this study. Time from explantation (p0) of the tumor samples to the first subcultivation step (p1) differed between the primary cell lines (22 ± 13 days) and the short‐term primary cell cultures (32 ± 23 days). This difference was not significant (*U*‐test, *P* = .17). The primary cell lines expanded continuously with a doubling time of approximately 2 to 14 days (Figure 1B and Table 2). Five of the 16 glioblastoma‐derived primary cell lines grew fast with a doubling time of 2.1 days (±0.3 day); seven grew in the medium with a doubling time of 5.4 days (±1.3 days), and the remaining four were slow growing (mean 11.4 ± 2.5 days). The three commercially available glioblastoma cell lines (A‐172, LN‐229, U‐251 MG), which were used as tumor specific comparison for immunohistochemistry, grew with a mean doubling time of 1.7 ± 0.2 days (Figure 1B).

**TABLE 1:** Clinical and histopathological data of the glioblastoma patients corresponding to the 16 established pCL

pCL	Gender	Age	Diagnosis	Location	MGMT promoter status	GFAP	p53	Ki‐67
11 029	M	43	Glioblastoma, IDH‐wildtype	Right occipital lobe	Methylated	Positive	3%	20%‐30%
11 030	F	71	Glioblastoma, IDH‐wildtype	Right temporo‐parietal lobe	Methylated	Positive	5%	20%
11 045	M	62	Glioblastoma, NOS	Left temporal lobe	Unmethylated	Positive	30%	20%‐30%
11 068	M	68	Glioblastoma, NOS	Right temporal lobe	Unmethylated	Positive	<1%	10%
11 091	F	79	Glioblastoma, NOS	Left fronto‐parietal lobe	Methylated	Weak	Negative	15%
12 040	M	61	Glioblastoma, IDH‐wildtype	Right temporal lobe	Unmethylated	Focal	5%	50%
12 041	F	56	Glioblastoma, IDH‐wildtype	Left parieto‐occipital lobe	Methylated	Positive	50%	50%
12 046	F	70	Glioblastoma, IDH‐wildtype	Right temporo‐occipital lobe	Unmethylated	Focal	10%	10%
12 058	M	77	Glioblastoma, IDH‐wildtype	Left frontal lobe	Unmethylated	Positive	1%	10%
12 059	F	66	Glioblastoma, IDH‐wildtype	Left temporal lobe	Methylated	Focal	50%	50%
12 060	M	50	Glioblastoma, IDH‐wildtype	Left frontal lobe	Methylated	Positive	60%‐70%	20%‐30%
12 073	M	91	Glioblastoma, IDH‐wildtype	Right frontal lobe	Unmethylated	Positive	10%‐20%	20%‐30%
12 075	F	56	Glioblastoma, IDH‐wildtype	Right frontal lobe	Methylated	Positive	5%	20%
12 079	F	68	Glioblastoma, IDH‐wildtype	Left parieto‐occipital lobe	Methylated	Positive	5%	20%
13 019	M	79	Glioblastoma, IDH‐wildtype	Left frontal lobe	Methylated	Focal	20%‐30%	30%
13 025	M	62	Glioblastoma, IDH‐wildtype	Left temporal lobe	Methylated	Positive	50%	30%‐40%

Abbreviations: F, female; GFAP, glial fibrillary acidic protein; IDH, isocitrate dehydrogenase; M, male; MGMT, O6‐methylguanine‐DNA‐methyltransferase promoter methylation status; NOS, not otherwise specified; pCL, primary cell lines.

**TABLE 2:** Summarized characterization results for the pCL and cGCL grouped according to GFAP expression

GFAP expression	Cell line #	Growth characteristics and morphology					Immunocytochemical characterization							
		Doubling time (days)	Layer morphology	Cellular shape	Migration	Colony formation	GFAP	S100B	TUBB3	VIM	NES	SOX2	MKI67	TE7
High	12 059	5.8	Focal growth	Pleomorphic	Single cells	No	5	3	5	5	5	2	3	0
	11 068	3.5	Focal growth	Pleomorphic	Single cells	Weak	4	3	5	4	4	1	0	1
	12 075	13.6	Focal growth	Pleomorphic	No	No	4	4	5	5	5	2	4	1
	**U‐251 MG**	1.4	Monolayer	Spindle‐form	Single cells	Strong	4	4	5	1	1	0	4	0
Medium	11 029	6.6	Monolayer	Polygonal	Collective	Strong	3	5	5	5	5	5	5	0
	13 019	1.6	Monolayer	Spindle‐form	Collective	Strong	3	5	5	5	5	4	4	0
	11 045	2.3	Focal growth	Pleomorphic	Single cells	Weak	3	5	4	4	4	4	2	2
	12 041	2.2	Focal growth	Polygonal	No	Strong	3	4	5	4	4	0	1	0
	12 079	2.3	Monolayer	Spindle‐form	Single cells	Weak	3	4	4	5	4	2	4	1
	12 073	4.2	Monolayer	Spindle‐form	Collective	Strong	3	5	4	5	2	1	3	0
	13 025	4.7	Focal growth	Polygonal	Single cells	Weak	3	4	4	5	2	1	2	1
	11 091	13.4	Focal growth	Polygonal	Single cells	No	3	2	4	4	1	0	2	1
Low	12 060	6.6	Focal growth	Pleomorphic	Collective	Weak	2	5	4	5	1	1	2	0
	12 040	6.7	Focal growth	Pleomorphic	Single cells	No	2	4	5	5	3	0	1	1
	12 058	8.5	Monolayer	Pleomorphic	Collective	Weak	2	4	5	4	4	1	3	1
	12 046	10.3	Monolayer	Pleomorphic	No	No	2	4	4	5	3	0	0	2
No	**LN‐229**	1.9	Monolayer	Spindle‐form	Collective	No	0	3	5	5	4	0	3	0
	11 030	1.8	Monolayer	Polygonal	Collective	Weak	0	0	5	3	0	0	2	1
	**A‐172**	1.8	Monolayer	Polygonal	Collective	Strong	0	0	5	2	0	0	3	0

*Note*: The three cGCL are shown in bold. In immunocytochemical characterization, the number of stained cells was scored as follows: 0, no expression; 1, ≤5% of cells; 2, ≥5% of cells; 3, ≥25% of cells; 4, ≥50% of cells; 5, ≥75% of cells.

### 3.2 Morphological classification and growth characteristics

Morphological classification of the established glioblastoma‐derived primary cell lines was performed to identify the cytomorphological diversity expected in the glioblastoma “multiforme” cell lines. For this purpose, morphological criteria of the cell layer and the cell shape were recorded. In addition, colony formation and migratory behavior of the primary cell lines were investigated (Table 2).

**FIGURE 2:**
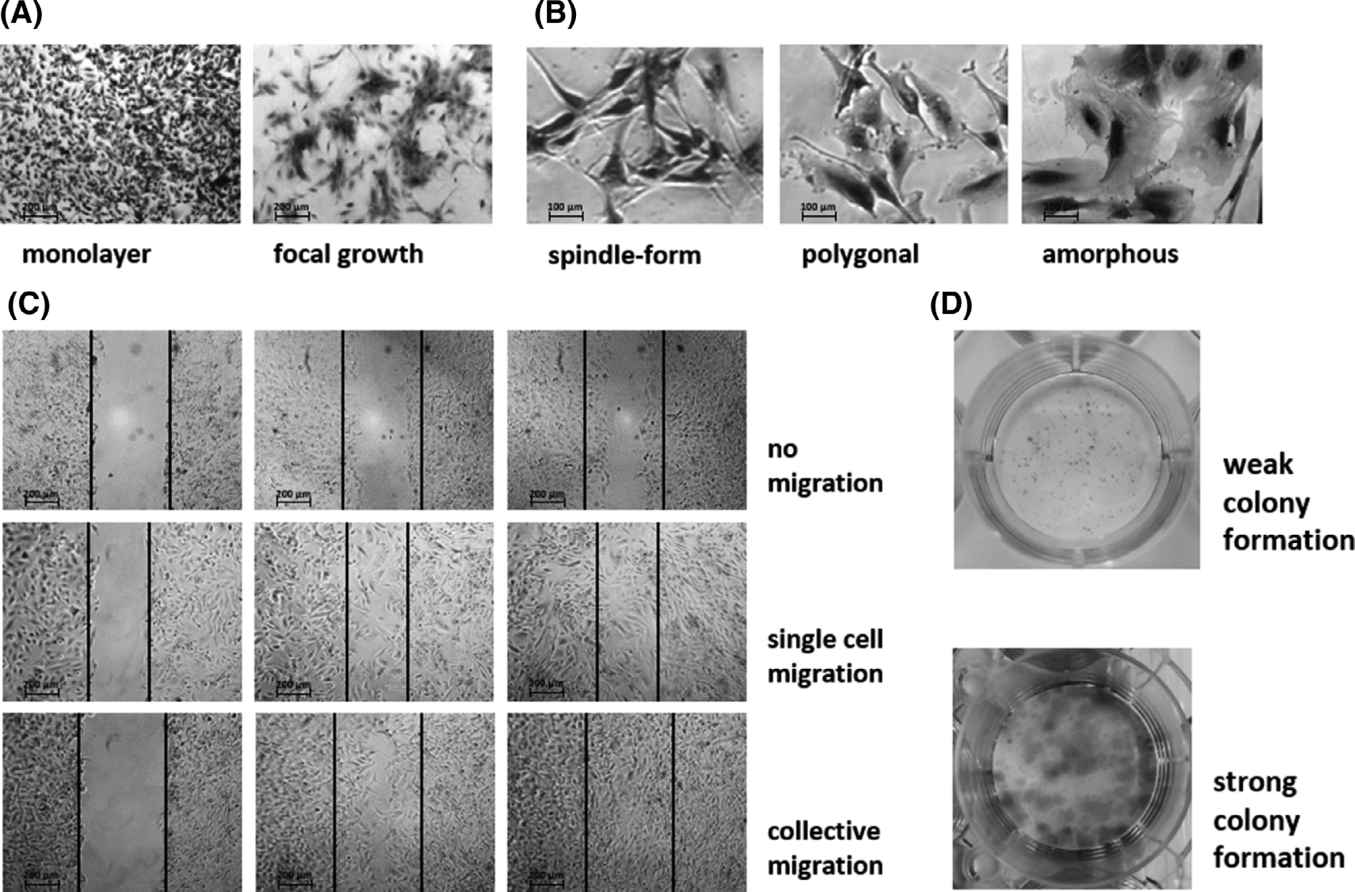
Examples of phase contrast images of cytomorphological characteristics (2.5‐fold). A, Layer morphology. B, Cellular shape. C, Migratory behavior. Example images at 0, 12, and 24 hours are shown. D, Colony formation

It was noticeable that the adherent cell layers of the outgrowing primary cell lines developed into two growth types (Figures 1C and 2A): (a) monolayer with cells growing side by side (7/16) and (b) focal growth, where the cells grew in islets connected by cellular protrusions (9/16). After reaching confluence, in the monolayer cultures, the cells grew on to multilayered cultures or formed cell clusters even under serum conditions. Cell lines with a focal growth tended to form cell clusters based on these foci, but only reached a maximum confluence of 75% to 90%. The commercial cell lines A‐172, LN‐229, and U‐251 MG grew as a monolayer. The primary cell lines exhibited three different main cellular shapes during cultivation (Figures 1D and 2B): (a) spindle‐form (3/16), (b) polygonal (5/16), and (c) amorphous with cellular protrusions (8/16). In addition, in most of the primary cell lines, dendritic‐like cells and small spindle‐form cells were visible. The cells of the cell lines LN‐229 and U‐251 MG showed a spindle‐form cell shape, and the cells of the cell line A‐172 were polygonal. All morphological groups had been described for glioblastoma cells cultivated adherently.^34^

To find out if the cells were able to migrate on a solid substrate and to grow out from single cells to a cell mass without cell‐cell contacts, the migratory behavior and the colony formation capability of the established primary cell lines were analyzed. According to their migratory behavior, it was possible to divide them into three groups (Figures 1E and 2C): (a) no migration (3/16), (b) single cell migration: single cells migrate into the scratch (7/16), and (c) collective migration: cells migrate side by side from the edge of the scratch to its center (6/16). The cell lines were migrating as single cells (U‐251 MG) or collectively (A‐172, LN‐229).

Analyzing colony formation, in 11/16 cases, the cells were able to proliferate without cell‐cell contacts. The colony formers were divided into two groups (Figures 1F and 2D): (a) weak colony formers: the proliferating cells were uniformly spread over the surface of the culture dish, forming small colonies (7/16) and (b) strong colony formers: distinct outgrowth to large colonies (4/16). The cell lines A‐172 and U‐251 MG were strong colony formers. LN‐229 did not form single colonies under these conditions; the cells had spread evenly across the entire well.

### 3.3 Immunocytochemical characterization

**FIGURE 3:**
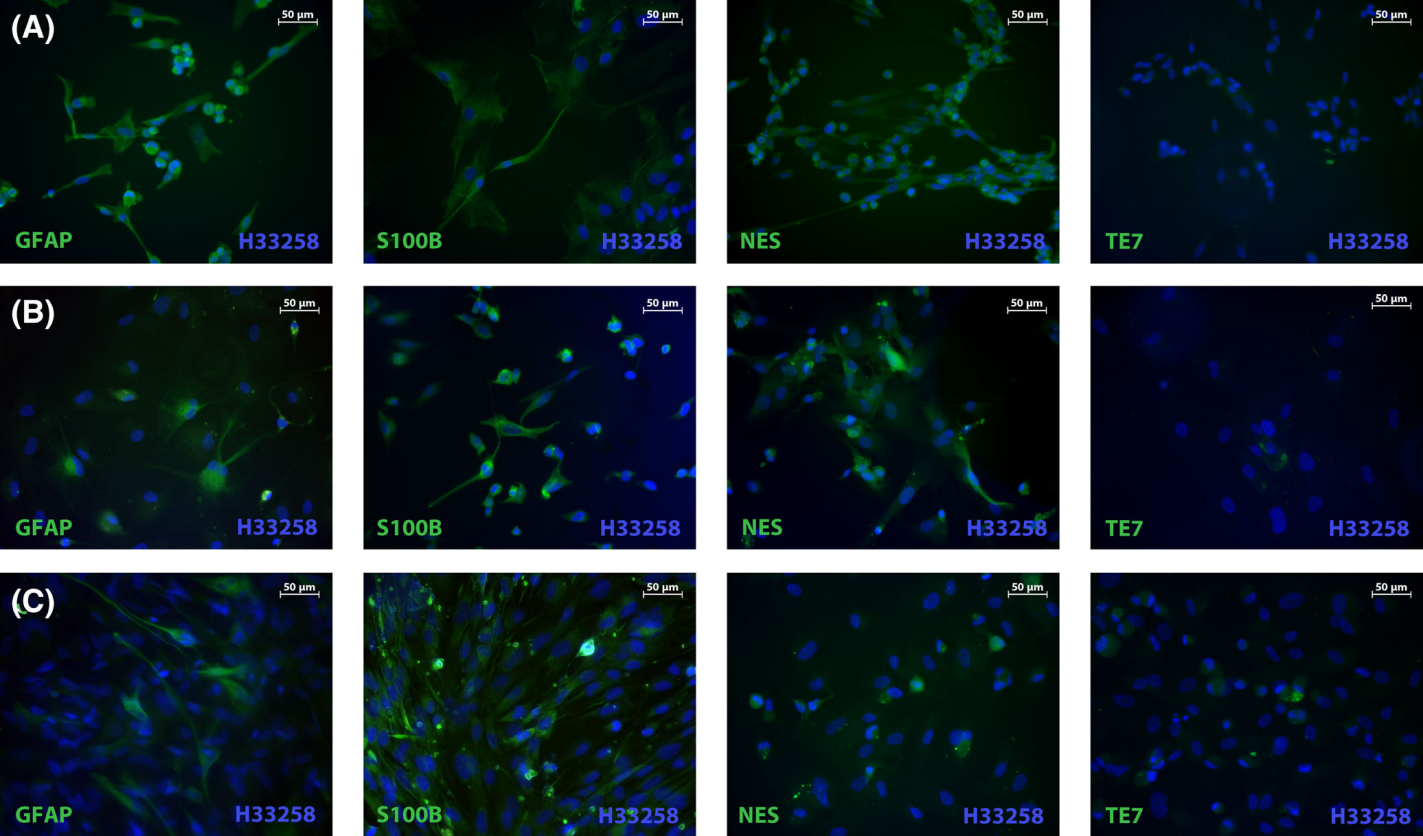
Immunocytochemical staining of pGCL. Examples for specific staining of GFAP, S100B, NES, and TE7 (green, FITC) in each GFAP expression group are given. Cell nuclei were counterstained with H33258 (blue). Magnification ×200. A, High GFAP expression group, B, medium GFAP expression group, and C, low GFAP expression group

**FIGURE 4:**
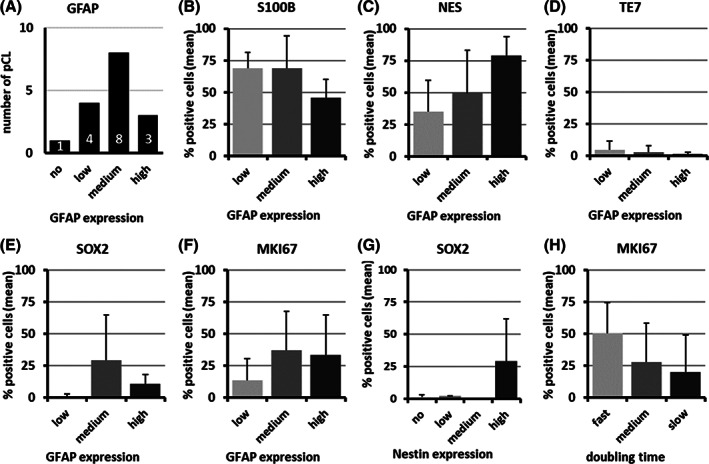
Immunocytochemical characterization of the primary glioblastoma cell lines (pGCL). A, GFAP expression in the cultivated primary glioblastoma cell lines. B‐F, Positive stained cells (mean percentage) in pGCL grouped by GFAP expression. B, S100B, C, NES, D, TE7, E, SOX2, F, MKI67. G, SOX2 positive stained cells (mean percentage) in pGCL grouped by Nestin expression. H, MKI67 positive stained cells (mean percentage) in pGCL grouped by doubling time

To specify staining criteria for the definition of primary glioblastoma cell lines and to ascertain whether the glioblastoma‐derived primary cell lines have similarities to glioblastoma tissue, we undertook an immunocytochemical staining for markers used in glioma diagnosis (Table 2 and Figures 3 and 4). The marker expression was compared to the commercially available glioblastoma cell lines (cGCL) A‐172, LN‐229, and U‐251 MG. Only U‐251 MG was immunopositive for GFAP and, in U‐251 MG and LN‐229, an expression of S100B was detectable. A172 showed neither GFAP nor S100B. Fifteen out of the 16 primary cell lines (94%) were characterized, retained the expression of GFAP and S100B as reliable markers of astrocytic or glial cells, and were defined as primary glioblastoma cell lines (pGCL). Only in one primary cell line, no GFAP and S100B expression was detectable. It had to be defined as nonglioblastoma cell line based on the validation criteria. However, the proportion of GFAP‐expressing cells varied markedly in the primary glioblastoma cell lines (Figures 3 and 4A): in 3/15 pGCL, it was high (>50%), in 8/15 pGCL, medium, and in 4/15 pGCL, low (≤10%). S100B expression was higher compared to GFAP. In 12/15 pGCL, it was high (>50%), in 2/15 pGCL, medium, and in only one pGCL, low. With increasing GFAP expression, the S100B expression decreased (Figure 4B, not significant). In 4/15 pGCL with a low GFAP expression, a low expression of NES (Figure 4C), SOX2 (Figure 4E), and MKI67 (Figure 4F) could be observed.

All pGCL displayed a strong positive staining signal for TUBB3 and VIM. Thirteen of the 15 pGCL were immunopositive for the malignancy marker NES, and of these, a further six were SOX2 positive (Figure 4G). Eleven of 15 pGCL expressed MKI67 as a proliferation marker. The expression is highest in the cell lines with a high doubling time (Figure 4H). Accordingly, none of the pGCL was classified as senescent. The analysis of the fibroblast marker TE7 revealed in 7/15 pGCL an immunopositivity in <1% of the cells and only in two pGCL, a low expression (in ≤10% of the cells, Figure 4D).

**FIGURE 5:**
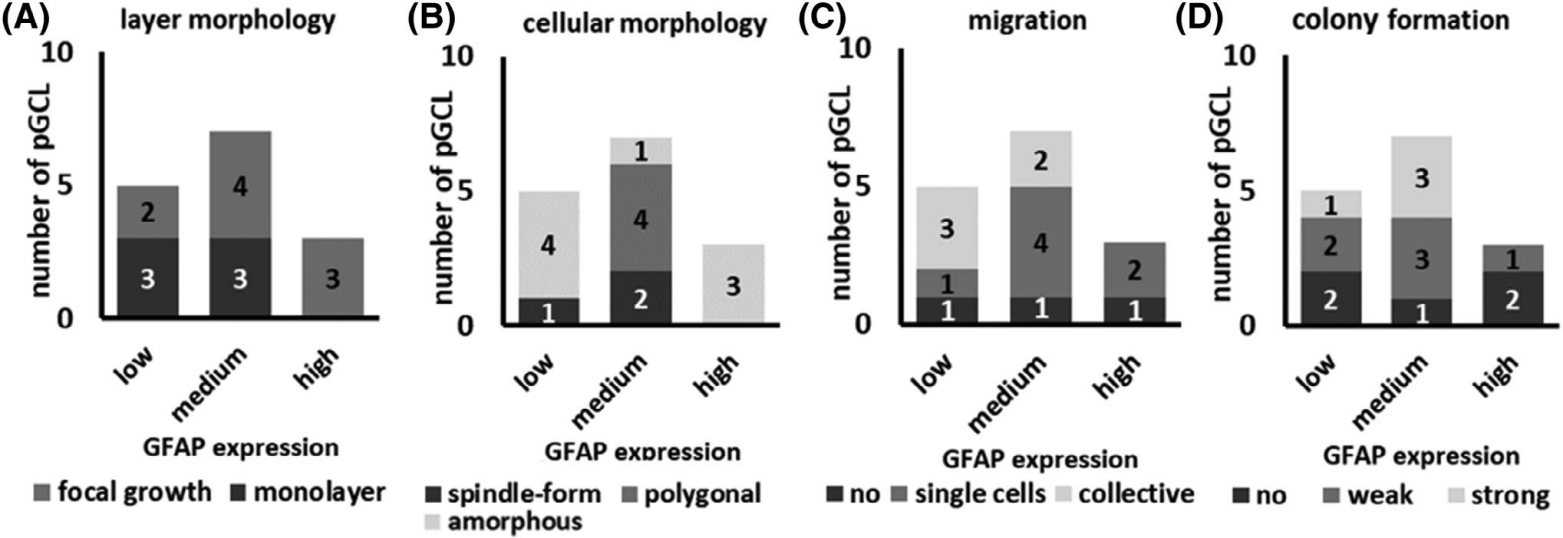
Distribution of morphological characteristics of the 15 pGCL between the three GFAP expression groups. A, Cell layer morphology. B, Cellular phenotypes. C, Migratory behavior. D, Ability to form colonies

The cytomorphological parameters defined for the primary glioblastoma cell cultures were analyzed in dependence of the GFAP expression strength to find possible associations (Figure 5). There were neither significant differences nor correlations in cytomorphological parameters between the three GFAP expression groups.

## 4 DISCUSSION

In vitro models are an important tool in investigating the cellular and molecular biology of malignant gliomas as well as specific treatment options for patients. Primary glioblastoma cell lines provide an excellent material source for all types of studies due to the available number of tissue samples from differing individual glioblastomas. Compared to spheroid cell cultures, these primary cell lines offer the advantage that the cells can be expanded more strongly. Primary cell lines can be converted into spheroid cultures by changing the cultivation conditions if necessary. Therefore, it is important to establish cell lines from individual glioblastoma samples in order to cover a broad spectrum and to ensure that the heterogeneity of the tumor entity is represented.^35^, ^36^, ^37^ Defined characterization criteria and validation guidelines for the establishment of primary cell lines from tumor material are mandatory. Passaging the cell lines as little as possible is preventing genetic or epigenetic alterations and thus keeping them close to the original tumor.^38^, ^39^

The biological properties of the primary glioblastoma cell lines established in this study were investigated to characterize the cellular behavior in vitro and to further determine whether correlations exist between the immunocytochemical, cytomorphological, and physiological parameters of the cell lines. Most cell lines contained cells that showed a typical morphology with amorphous cell bodies with protrusion, while also dendritic‐like cells and spindle‐form cells in the cultures were visible. In most cases, these cell lines were growing in focal cell clusters. The glioblastoma is characterized by its inhomogeneous and diverse (hence: “multiforme”) appearance. These morphological features as first indication of the presence of glioblastoma “multiforme” cells in culture have been described in different studies^25^, ^34^, ^40^ and observed in the cell lines established in this study. The observed morphological variability results from the involvement of different malignant and nonmalignant cell types in the tumor. Neftel et al^41^ described a set of cellular states for malignant glioblastoma cells: neural progenitor‐like (NPC‐like), oligodendrocyte‐progenitor‐like (OPC‐like), astrocyte‐like (AC‐like), and mesenchymal like (MES‐like) states. The associated nonmalignant cells include tumor‐associated macrophages, endothelium, and astrocytes.^42^ Since we could detect markers for the astrocytic and oligodendroglial lineage as well as progenitor cell signatures in our cell lines, it can be concluded that under the described cultivation conditions, our primary glioblastoma cell lines are composed of different malignant cell types. However, it can be assumed that nonmalignant cells cannot proliferate in the serum‐based culture medium used. The sole determination of cytomorphological parameters is not sufficient for the identification of pGCL. For this reason, it is imperative to find alternative methods that are easy to handle in order to identify pGCL uniquely. A potential technique is the immunocytochemical staining using a marker panel to detect glioblastoma‐specific protein expression. Further, in several studies, the colony‐forming behavior of glioma cell lines and the influence of different substances on colony formation were demonstrated.^43^, ^44^ In addition, the scratch assay has been used in a number of studies investigating the impact of drug treatments on the migratory ability of glioma cell lines.^45^, ^46^, ^47^ Both methods, colony formation assay and scratch assay, were carried out in their simplest form in this study. Theoretically, the tumor cells should migrate and form colonies. The results showed that not all primary glioblastoma cell lines were able to migrate (81%) and to form colonies (69%) in the present experimental setting, indicating the importance of analyzing colony formation and migratory behavior for characterization of glioma cells in vitro using different methods for a more reliable informative value. For intracranial tumor cells, it is possible that in the used in vitro system, not all needed stimuli, inducing colony formation and migration, are present, addressing the role of the environment. For this reason, a potential alternative technique is the inoculation of mouse brain slices with the cultivated pGCL to analyze both colony formation and migratory behavior. Further, tumor spheroid‐based migration assays can be used.

For immunocytochemical characterization, antibodies against glial markers, neuronal, and neural precursor markers, as well as malignancy markers, were selected (GFAP, S100B, TUBB3, VIM, NES, and SOX2). To identify the established primary cell lines as glioblastoma cell lines, an immunopositivity for both glial markers GFAP and S100B was defined. GFAP is a protein involved in the structure and function of the cytoskeleton and is commonly used as an astrocytes marker: its expression is increased following brain damage or during degeneration of the central nervous system.^48^ In glioblastomas, this antigen is strongly expressed in the cytoplasm of the tumor cells.^20^ Although predominant among the water‐soluble brain proteins, S100B is also found in a variety of other tissues. It is expressed in schwannomas, ependymomas, gliomas, and almost all benign and malignant melanomas and their metastases.^49^, ^50^ Since healthy cells of the brain parenchyma cannot be cultured under these cultivation conditions, GFAP and S100B can be used as markers for tumor cells in primary glioblastoma cell lines. The ubiquitous expression of VIM as a malignancy marker in the investigated pGCL, which identifies the cultured cells as tumor cells, underlines this. Applying the defined validation criterion, expression of both markers GFAP and S100B, 15 primary glioblastoma cell lines (pGCL) could be establish from 34 intraoperative glioblastoma samples processed in this study, resulting in a total efficiency of 44%. Only one primary cell line was excluded due to the lack of GFAP and S100B expression. The proportion of GFAP positive cells in the primary glioblastoma cell lines, however, varied considerably. Besides, in the 4/15 pGCL with a low GFAP expression, a reduced expression of malignancy and proliferation markers could be observed compared to the other pGCL. In addition, the expression of TE7 is highest in this group. Although these expression differences are not statistically significant due to the small group size, GFAP low expressing group could be excluded for functional analyses on glioblastoma cell lines based on these findings. Therefore, the expression of malignancy and proliferation markers in combination to the proportion of GFAP and S100B expression should be assessed to define the usefulness of self‐established glioblastoma cell lines for further experiments.

The commercially available glioblastoma cell lines A‐172, LN‐229, and U‐251 MG were analyzed as a comparison group for the established pGCL. Only U‐251 MG showed GFAP and S100B expression as expected. Surprisingly, GFAP was not detectable in A‐172 and LN‐229 cell lines. In U‐251, MG, and LN‐229, the expression of S100B was detectable as reported.^34^ Only for A‐172, the negative expression results for GFAP could be verified by the literature.^51^ According to the defined validation criterion, only U‐251 MG is still a glioblastoma cell line. A reason for these results could be the long period of time since the establishment of the commercial cell lines and therefore the associated accumulations of genetic and epigenetic changes. Our findings underline the critical use of established cell lines and the importance of using primary cell lines in investigating tumor biology or for functional analyses such as response prediction.

TUBB3 and VIM have been identified in some human glioma cell lines before.^52^, ^53^ VIM as type III intermediate filament protein, a major cytoskeletal component, has no diagnostic value in brain tumors. In GBM, there was a positive but highly variable immunohistochemical staining of cytoplasm and processes of cells of different kinds reported.^54^ It was identified as an independent prognostic factor for high‐grade glioma patients.^55^ TUBB3 as a microtubule protein mainly expressed in cells of neuronal origin has been revealed as overexpressed in many cancers including gliomas.^56^ In gliomas, TUBB3 expression seems to correlate with an increased malignancy, high proliferative rates, and poor prognosis.^57^, ^58^ All pGCL established in this study displayed a strong immunopositivity for VIM and TUBB3. In addition to the astrocytic markers GFAP and S100B, these two markers can be used to confirm the malignancy of established cell lines.^16^, ^59^, ^60^

The verified protein expression of the progenitor markers NES and SOX2 in the adherent primary glioblastoma cell lines established here is indicating that the cells retained at least the ability to dedifferentiate, if not potential stem cell properties. Only pGCL positive for NES showed an expression of SOX2. These pGCL also expressed MKI67 as a proliferation marker.

To exclude a selection for fibroblasts in the cell cultures, all pGCL were stained for the fibroblast marker TE7^61^ and revealed, in seven of the pGCL, a slightly positive, and in two pGCL, a positive signal. These data show that the primary glioblastoma cell lines contain a mixture of different cell types, but that none of the cultivated pGCL had to be identified as a fibroblast cell line.

Some of the established pGCL could be cultivated over passage 30 (data not shown). This led to a clonal selection and therefore the standardization of the phenotype. These cell lines largely lost GFAP expression. It only remained in individual cells, as indicated by the literature before.^52^, ^58^ However, these cell lines continued to express S100B, TUBB3, and VIM in most of the cells, together with NES in a part of the cells, revealing a neural lineage specification and differentiation.

## 5 CONCLUSION

In conclusion, these findings indicate that serum‐based cultivation enables routine expansion of glioblastoma cells and establishment of defined primary glioblastoma cell lines, maintaining heterogeneous cell types over the first passages. The accurate characterization and validation of the cell lines is a mandatory step in the implementation of in vitro glioblastoma research. In principle, the characterization should be performed assessing the expression profiles of the astrocytic markers GFAP and S100B. Using NES as a marker, a proportion of cell lines with potential in terms of malignancy can be determined. Despite the heterogeneous nature of primary cell lines and the broad range of morphological phenotypes displayed by the cells, morphological assessment,and determination of generation time are also important for characterization of self‐established primary cell lines. The migratory behavior and ability to form colonies of the primary cell lines provide additional information for in vitro glioblastoma research.

## CONFLICT OF INTEREST

The authors declare no conflicts of interest.

## ETHICAL STATEMENT

This study was approved by the local human research ethics committee of the Friedrich Schiller University Jena (Ethik‐Kommission der Friedrich Schiller Universität Jena: AZ 3253‐10/11) and was performed in accordance with current legislation and the ethical standards laid down in the 1964 Declaration of Helsinki and its later amendments. All patients gave their written informed consent to study participation.

## AUTHOR CONTRIBUTIONS

**Susanne Grube:** Data curation; formal analysis; investigation; methodology; project administration; validation; visualization; writing‐original draft; writing‐review and editing. **Diana Freitag:** Data curation; formal analysis; investigation; methodology; validation; visualization; writing‐original draft; writing‐review and editing. **Rolf Kalff:** Conceptualization; resources; writing‐review and editing. **Christian Ewald:** Conceptualization; data curation; project administration; resources; supervision; writing‐review and editing. **Jan Walter:** Conceptualization; data curation; project administration; resources; supervision; visualization; writing‐original draft; writing‐review and editing.

## Data Availability

All data analyzed during this study are included in this published article. The raw datasets used for analysis in the current study are available from the corresponding author on reasonable request.
